# Age-related morphological changes of the pubic symphyseal surface: using three-dimensional statistical shape modeling

**DOI:** 10.1038/s41598-024-84168-8

**Published:** 2025-01-02

**Authors:** Yuyoung Kim, Kang-Woo Lee, Sookyoung Lee, Eun Jin Woo, Kyung-Seok Hu

**Affiliations:** 1 Division in Anatomy and Developmental Biology, Department of Oral Biology, Human Identification Research Institute, BK21 FOUR Project, Yonsei University College of Dentistry, Seoul, 03722 Republic of Korea; 2 School of Mechanical and Robotics Engineering, Gwangju Institute of Science and Technology, Gwangju, 61005 Republic of Korea; 3 Division of Forensic Medical Examination, National Forensic Service, Wonju, 26460 Republic of Korea; 4 Department of History, College of Liberal Arts, Sejong University, Seoul, 05006 Republic of Korea

**Keywords:** Pubic symphyseal surface, Age-related morphological changes, Age-at-death estimation, Statistical shape modeling, Human variation, Forensic anthropology, 3-D reconstruction, Bone, Statistical methods, Biological anthropology

## Abstract

Computational analysis of the pubic symphyseal surface is widely used for accurate age estimation, offering quantitative precision through the detection of subtle morphological changes. However, these methods often lack insights into the underlying morphological changes across different age groups. To bridge this gap, the study utilizes statistical shape modeling (SSM), a versatile tool capable of describing diverse morphological variations within populations. This study aimed to elucidate the direction and extent of these morphological changes, identify the contributing factors, and pinpoint key variations crucial for distinguishing between age groups. Computed tomography (CT) scans of 252 subjects from the National Forensic Service of South Korea were employed, subjected to preprocessing and landmark-based alignment. Through this approach, the study visualized and validated significant age-related morphological changes and highlighted the shape variations essential for differentiating between consecutive age groups. This study holds significance in elucidating the intricate nature of age-related morphological changes in the pubic symphyseal surface. Thus, these findings can serve as valuable cornerstones for enhancing age-at-death estimation techniques in forensic anthropology.

**Supplementary Information:**

The online version contains supplementary material available at 10.1038/s41598-024-84168-8.

## Introduction

Visual analysis of the pubic symphyseal surface has long been a cornerstone of age-at-death estimation in the field of biological anthropology^1–3^. Various standard methods based on phases or components have been developed^4–7^, among which the method devised by Suchey and Brooks has dominated the other methods in terms of application frequency^3,8^. However, numerous limitations have been highlighted, including the subjectivity of visual assessment, significant age overlap between groups, and low accuracy in the estimation of older subjects^9–11^. This has led to a point where there is no choice but to estimate the age of unidentified individuals into broad categories, such as young, middle, and old adults.

Efforts to overcome these limitations have resulted in the adoption of computed tomography-based mathematical approaches to automate age estimation of the pubic symphyseal surface for quantitative and highly accurate result^12–18^. Initially, mathematical methodologies are employed to calculate convexness and concaveness based on the observation that the curvature pattern of the pubic symphyseal surface becomes blurred, and the surface flattens with age^12,13^. Subsequently, analyses utilized bending energy through the thin plate splines (TPS) algorithm or the variance- based SAH score suggested by Slice and Algee-Hewitt to assess variations in surface morphology^14–17^. However, these studies had limitations such as underestimating the age of older individuals^15–17^. More recently, research utilizing algorithms such as Simple Automated Symphyseal Surface-based (SASS) and Advanced Automated Neural Network-grounded Extended Symphyseal Surface-based (AANNESS), which incorporate various population groups and mixed sexes, has demonstrated higher accuracy in age estimation^18^. These studies have enabled the detection of subtle morphological changes that are difficult to discern with the naked eye, increasing age estimation accuracy.

However, the primary focus of computational methods is the detection and evaluation of subtle morphological differences across age groups, thereby overlooking the fundamental age-related changes occurring on the pubic symphyseal surface. In other words, previous studies could not point out the morphological change that computational methods employed to discriminate between different age groups, which is called the “Blackbox”. Castillo et al. investigated morphological changes on the pubic symphyseal surface across different age groups; however, they did not provide clear indications of the direction or extent of these changes^19^. Therefore, our study utilized statistical shape modeling (SSM) to reveal morphological changes in the pubic symphyseal surface with aging. The SSM is commonly used to describe the diverse morphological variations within a shape group^20,21^. Due to its versatility, the SSM has been applied in various biological studies, including confirming variations in anatomical structures^22–24^, developing computed tomography (CT) and magnetic resonance imaging (MRI) segmentation methods^20,25,26^, and assessing pathological morphologies^27,28^. Recently, SSM has also been used in forensic anthropology, particularly for evaluating sexual dimorphism^21,29^ age at maturity^30,31^, and reconstructing intact bones^32^. Thus, this study aimed to achieve the following objectives by utilizing SSM: visualizing the morphological changes of the pubic symphyseal surface with aging, estimating reasonable factors of the changes, and identifying key morphological variations for distinguishing between consecutive age groups.

## Method

### Data acquisition

A total of 641 CT images of adults whose bodies were subjected to forensic examination by the National Forensic Service of South Korea between 2020 and 2021 were utilized. Individuals of South Korean nationality with available premortem information, including sex and age, were included. After a thorough inspection of the data, pubis bones with evident pathology and damage were excluded from the analysis. 18 males and 18 females in each age group, ranging from their twenties to eighties, were randomly selected, resulting in 252 samples. A detailed description of age distribution is provided in Table 1. Approval of the use of CT data (Protocol Number 2-2023-0071) was obtained from the Institutional Review Board of Yonsei University, College of Dentistry, Seoul, South Korea, which waived the requirement for informed consent from all subjects and/or their legal guardians. All methods were performed in accordance with relevant guidelines and regulations.

**Table 1:** Age distribution for each age group in the sample data.

Age groups	Mean age ± Standard deviation		
	Male	Female	Total
20 s	23.8 ± 2.6	23.9 ± 2.9	23.9 ± 2.7
30 s	34.8 ± 2.6	34.2 ± 3.0	34.5 ± 2.8
40 s	45.4 ± 3.0	45.4 ± 2.8	45.4 ± 2.9
50 s	53.8 ± 2.5	55.1 ± 2.7	54.5 ± 2.7
60 s	63.2 ± 2.5	63.4 ± 2.4	63.3 ± 2.4
70 s	73.8 ± 2.5	73.9 ± 2.6	73.9 ± 2.5
80 s	82.8 ± 2.6	84.7 ± 3.2	83.8 ± 3.0

For analysis, the pubic symphysis was first extracted from whole-body CT data, which were stored in digital imaging and communication in medicine (DICOM) format. The 3D slicer (version 5.3.0, http://www.slicer.org) and Meshmixer (version 3.5. 474, https://meshmixer.com) were used to remove the soft tissues surrounding the pubic symphysis and to isolate and separate the left and right pubic bones. In this study, only the left pubic symphyseal surface was utilized for analysis under the assumption of bilateral asymmetry in the human skeleton^33^. Next, the extracted pubic symphysis was subjected to preprocessing. This is because the data obtained through segmentation may contain intensive noise or redundant mesh components that are irrelevant to anatomical features. Therefore, a preprocessing step is crucial to eliminate potential interference and prepare data suitable for shape analysis and modeling (Supplementary Fig. 1).

### Statistical shape modeling (SSM)

#### Initial alignment and region of interest (ROI) designation

The processed pubic data were first transformed into a suitable format for ShapeWorks (version 6.3.2, https://sciinstitute.github.io/ShapeWorks/6.3). For shape analysis, it was necessary to align shapes oriented in different positions and directions to ensure that only intrinsic shape variations were captured, independent of positional differences. Therefore, an initial alignment was required to arrange the data to obtain a consistent orientation. In our study, five landmarks—superior, inferior, ventral, dorsal, and center of the symphyseal surface—were manually designated on the sample data, and the Iterative Closest Point (ICP) algorithm was then applied to align these landmarks across 252 pubic symphysis samples (Supplementary Fig. 2A)^34^. Based on these five landmarks, thousands of vertices on each of the 252 pubic symphysis samples were aligned. Finally, the region of interest (ROI) was restricted to an area that extended 1 cm vertically from each margin of the articulation surface (Supplementary Fig. 2B).

#### Shape model construction

To construct SSM, the vertices of each dataset must correspond to each other. To achieve this, ShapeWorks employs a particle-based surface-modeling (PSM) algorithm. PSM positions landmarks based on information entropy, strategically concentrating them in areas of greater shape variability^34,35^. In this study, 4096 landmarks were automatically designated on rigidly aligned data using PSM. In addition, Procrustes registration was applied alongside the PSM to further enhance the accuracy and consistency of landmark placement^36^, with translation, rotation, and scaling applied throughout this process.

#### Principal component analysis

Principal component analysis (PCA) was employed to capture shape variations within the dataset while preserving essential shape information^37^. During the PCA, the mean model of each age group was extracted, representing the average shape within the respective age groups. The mean model later served as a reference for comparing age groups, offering quantitative and visual descriptions of age-related morphological changes.

#### Shape model evaluation

Three commonly used metrics—compactness, generalization, and specificity—were employed to quantitatively evaluate the statistical shape model^35,38^. Compactness serves as an indicator of the efficiency of a shape model by assessing whether a wide range of morphological variations can be reproduced using a small number of principal components (PCs). Generalization evaluates the ability of the generated PC model to reconstruct shapes beyond the dataset used for analysis. This was assessed using the error value between the unseen and reconstructed data. Specificity evaluates the capacity of the PC model to produce reasonably shaped data randomly. This is considered ideal when the error value between the random-shape data and the closest sample data increases minimally as the number of PCs used increases.

#### Shape model comparison

By utilizing the *model to model distance* module of SlicerSALT (version 4.0.1, https://salt.slicer.org), the spatial displacement of the corresponding landmarks was depicted with vector arrows and heatmaps, illustrating morphological changes from the younger age group to the older age group. To determine whether the observed morphological changes were significant, Hotelling’s T-squared test was used (*p* < 0.05)^34^.

#### Shape-based classification

The accuracy of classifying age groups in a sequence based on morphological characteristics was assessed using the Classification Learner application in MATLAB (version R2023a, MathWorks). The response and predictor variables were the age group and PCA score of the combined dataset of two consecutive age groups, respectively. For instance, to analyze the differences between individuals in their 20 s and 30 s, PCA was performed on the 36 samples (18 from each age group). All available training algorithms in MATLAB were utilized, and the training was conducted using tenfold cross-validation. For the algorithm with the highest classification accuracy in each comparison, the most prominently used PC for distinguishing between two consecutive age groups was determined using an ANOVA-based feature-ranking algorithm.

## Result

### Principal component analysis

**Fig. 1:**
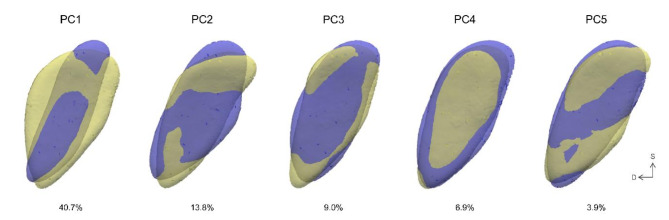
Shape variation explained by principal components 1 to 5. A model with a weight of − 2 standard deviations (violet) and one with + 2 standard deviations (yellow) are superimposed. Overlapping regions between the two models are shown in colors other than violet or yellow. Each PC captures one or more distinct morphological variations. In the orientation labels, ‘D’ indicates dorsal, and ‘S’ indicates superior.

Figure 1 illustrates the top five PCs derived through the PCA of 252 pubic symphyseal surface datasets. Each PC demonstrates distinct features: PC1 reflects the width of the symphyseal surface; PC2 shows whether the central portion of the margin protrudes ventrally or dorsally; PC3 highlights the sharpness of the inferior point combined with the straightness of the ventral or dorsal margin; PC4 reveals the bulginess of either the central or marginal sections of the symphyseal surface; and PC5 identifies whether the superior, central, or inferior third of the surface exhibits bulging.

### Shape model evaluation

**Fig. 2:**
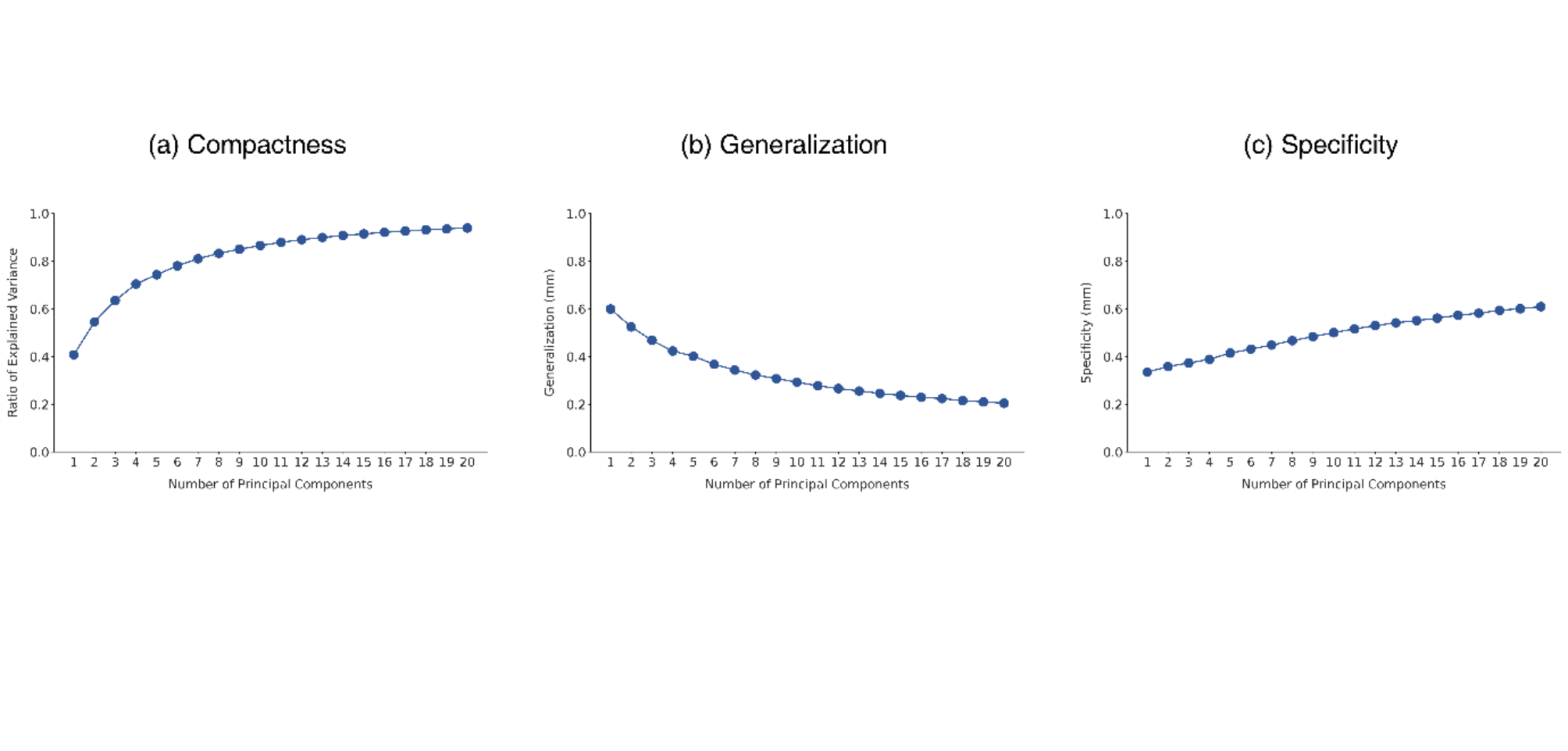
Shape model evaluation metrics. Compactness (**a**) demonstrates whether the PC model represents many shape variations with a small number of PCs. Generalization (**b**) evaluates the capacity to rebuild models that were not included in the sample data with error value between unseen data and rebuilt data. Specificity (**c**) assess ability of PC model to randomly produce reasonable data with error value between randomly rebuilt data and closet sample data.

Figure 2 illustrates the three quantitative assessments of the constructed statistical shape model. Compactness (Fig. 2a) indicates how efficiently the morphological variability can be represented. The top ten PCs capture approximately 85% of the shape variability in the pubic symphyseal surface data, whereas the top 20 PCs accounted for approximately 95%. Generalization (Fig. 2b) evaluates how effectively the PCA model reconstructs shapes beyond the data used in the analysis, with error values between the two. As evident from the results, increasing the number of utilized PCs led to a decrease in the error value from approximately 0.6 mm to 0.2 mm. Specificity (Fig. 2c) evaluates whether the PCA model can randomly generate a rational shape model with an error value between the random model and its closest model. An increase in the number of PCs utilized naturally leads to increased complexity; however, this increase should not be sharp. In this study, the specificity results demonstrated a minimal increase in error values of up to approximately 0.2 mm until PC20.

### Shape model comparison

**Fig. 3:**
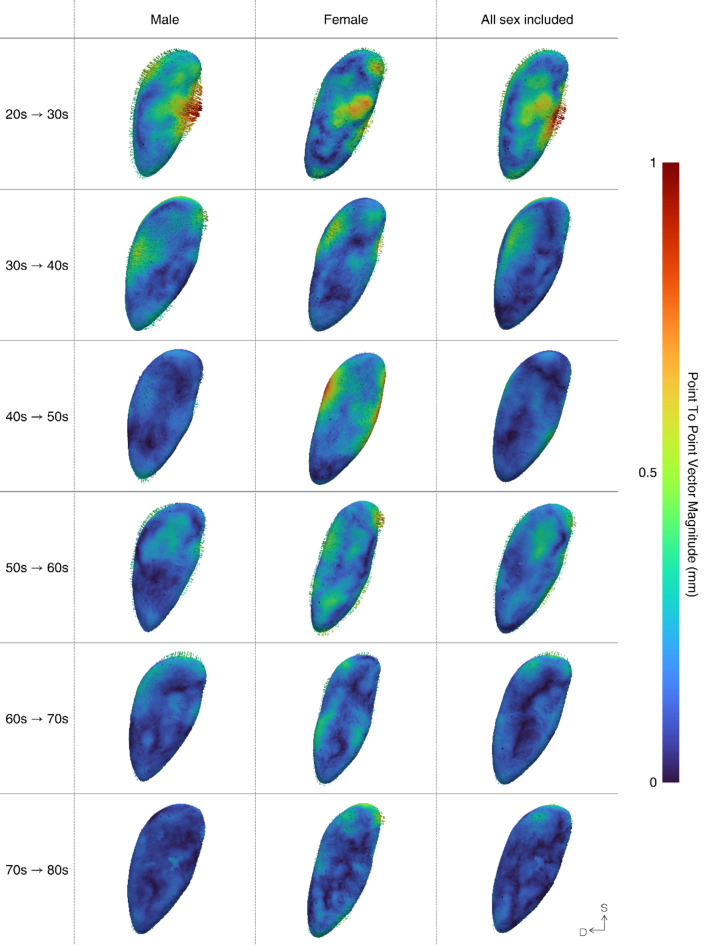
Three-dimensional visualization of the relative difference that the older age group pubic symphyseal surface exhibits compared to that of the younger age group. The extent of variation increases from blue to the most significant difference concentrated in red. Three-dimensional vector arrows depict both the direction and magnitude of these changes, with their size doubled for enhanced clarity. In the orientation, ‘D’ stands for dorsal and ‘S’ stands for superior.

**Fig. 4:**
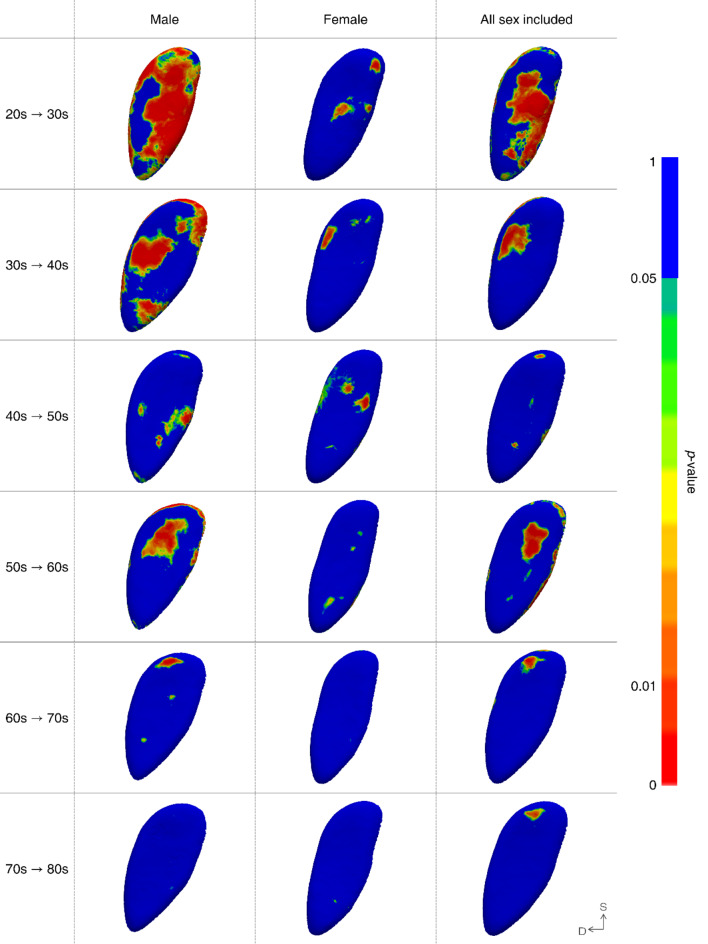
Regions exhibiting statistically significant morphological changes. Areas with p-values exceeding 0.05 are indicated in blue, while those below 0.05 are color-coded according to their significance level, with the most significant changes highlighted in red. In the orientation, ‘D’ stands for dorsal and ‘S’ stands for superior.

Through PCA, the mean pubic symphyseal surface models were extracted for each age and sex group, including combined sexes (Supplementary Fig. 3). Age-related morphological changes observed in the mean pubic symphyseal surface models for each age group were visualized (Fig. 3). Subsequently, the statistical significance of the morphological changes was validated (Fig. 4). For clarity, the sample data were categorized into three groups: young (YA), middle-aged (MA), and old adult (OA) groups, for analysis. In both males and females, the most dynamic morphological changes occurred in the YA, especially between the 20 s and 30 s age groups. For subsequent age transitions, the magnitude of change decreased, while a slight increase was observed in MA, i.e. in males in their 50 s to 60 s, and in females in their 40 s and 50 s. However, while males experienced a significant decrease in the rate of change with age, females exhibited a smaller decline in the rate of change across all age ranges except over 60 years of age. Substantial morphological changes were noted in males, whereas those in females were rarely apparent. Moreover, the focal points of morphological changes varied with age. In the YA group, changes in surface characteristics were predominantly observed, whereas alterations in the outline prevailed in the subsequent groups for both males and females. Moreover, when a shape model was constructed without sex separation, the pattern of change closely resembled that observed in males.

A detailed description is as follows: in the YA group, the pubic symphyseal surface exhibited a notable extension trend towards both the ventral and dorsal margins, irrespective of sex. Moreover, significant alterations were observed near the ventral margin in the 20 s–30 s age range, while in the 30 s to 40 s age range, substantial changes were noted near the dorsal margin. In males, a distinct alteration was observed near the center of the ventral margin during the transition from 20 to 30 s. Although morphological changes were evident in females, they were not statistically significant. In the MA group, both sexes experienced a slight increase in the magnitude of change, as previously mentioned. However, the pattern differed slightly, with males exhibiting statistically significant alterations in the superior half of the surface. In contrast, females displayed changes in both the ventral and dorsal margins, but without statistical significance. Finally, in the OA group, few substantial changes were observed in both males and females and statistically significant alterations were almost nonexistent.

### Shape-based classification

The classification algorithm with the highest accuracy and its corresponding accuracy within each age-sex group were determined by analyzing the PCA scores of sample data from two consecutive age groups using the MATLAB classification learner app (Table 2). For information on the algorithm models that demonstrated the highest accuracy for each group, please refer to Supplementary Table 1. The transition with the highest accuracy was observed from 20 to 30 s in both males and females. Additionally, there were slight increases in accuracy during specific transitions: from the 50 s to 60 s in males, from the 40 s to 50 s in females, and from the 70 s to 80 s in females. Moreover, except for the transition from males in their 70 s to 80 s and females in their 50 s to 60 s, the results indicated that considering sex separately for age classification resulted in a higher classification accuracy than analyzing all sex combinations.

**Table 2:** Performance of classification training.

Compared age groups	Accuracy (%)		
	Male	Female	Total
20 s → 30 s	83.3	83.3	77.8
30 s → 40 s	80.6	72.2	70.8
40 s → 50 s	66.7	75.0	65.3
50 s → 60 s	83.3	61.1	63.9
60 s → 70 s	77.8	66.7	58.3
70 s → 80 s	63.9	77.8	66.7

**Fig. 5:**
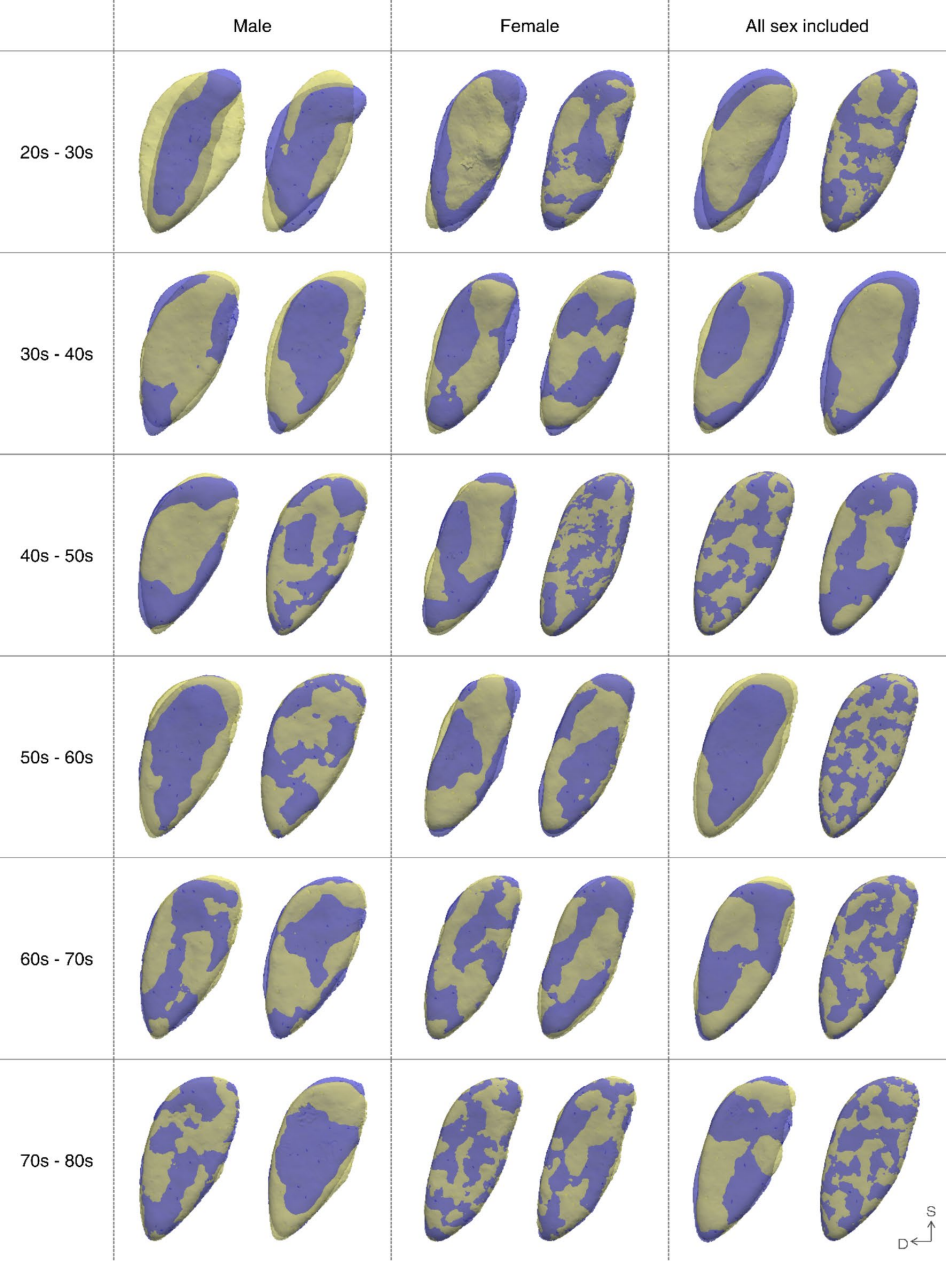
Two shape variations significant in classifying between two successive age groups. A weight of − 2 standard deviation (violet) model and a weight of + 2 standard deviation (yellow) model is superimposed. The overlapping between two models is colored neither in violet nor yellow. In the orientation, ‘D’ stands for dorsal and ‘S’ stands for superior.

Subsequently, shape variations crucial for age classification were verified. The top two significant shape variations for age classification were presented for each age-sex group, with the most prominent depicted on the left and the subsequent one on the right (Fig. 5). Further information on the percentages of explained variance for each age transition can be found in Supplementary Table 2. Generally, each PC encompasses two or more shape variations. Surface details were presented in all PCs across sexes and age groups, although their patterns were challenging to clearly define and appeared irregular. When males and females were not distinguished, surface details acted as one of the most significant factors in classifying age groups, except for the most significant PC for the 20 s-30 s age group, which showed a curve in the ventral or dorsal margin.

The significant shape variations in each age group were as follows: In the YA group, in addition to sex disparities, differences were noted between the 20 s and 30 s and 30 s to 40 s age transitions. During the transition from 20 to 30 s, males exhibited symphyseal surface width as the most pivotal factor in distinguishing between 20 and 30 s, followed by discerning curved areas along the ventral or dorsal margins. Conversely, among females, the foremost determinant was the extent of surface protrusion, along with the same factor observed as a secondary significant factor in males. The subsequent critical factor emerged as irregular surface characteristics. Notable components for distinguishing between the 30 s and 40 s appeared similar across the sexes, with the primary factors being the sharpness of the inferior point and the notch figure of the ventral margin. Subsequently, noteworthy PC-embedded information refers to the length of the pubic symphysis and surface details. In both the MA and OA groups, all significant PCs contained descriptions of surface details. As mentioned previously, these surface details manifested as irregular morphologies that could not be defined as standardized descriptions. However, in the OA group, the most critical PCs explained the more complex forms of surface variations.

## Discussion

### Principal component analysis

PCA reduces dimensionality by prioritizing axes with greater variance. High-ranked principal PCs are generally considered effective for capturing significant patterns within a dataset^39^. Consequently, many forensic anthropology studies utilizing PCA have relied on higher-ranked PCs to differentiate variables such as age or sex based on skeletal morphology.

However, the high-ranked PCs extracted from PCA primarily reflect the dominant morphological variations within a dataset, which often represent individual differences rather than providing direct indicators for age or sex classification. Instead, researchers highlight that lower-ranked PCs, though frequently dismissed as noise during dimensionality reduction, effectively capture subtle and localized patterns in the data that can be crucial for specific classification tasks^39–42^.

In this study, the higher-ranked PCs extracted from the PCA of 252 pubic symphyses (Fig. 1) did not consistently show significance for age group classification across all groups. As discussed later in the *Shape-Based Classification* section and as illustrated in Fig. 5, the shape variations significant for distinguishing between age groups include those represented by the higher-ranked PCs and a broader range of patterns. Supplementary Table 2 shows comparisons among older age groups revealed that shape variations with lower explained variance were more effective in distinguishing age groups. This finding suggests that in the pubic symphysis, subtle and localized patterns become more meaningful for age differentiation as age increases.

This study identifies shape variations by conducting PCA on datasets from two groups simultaneously. Expanding this approach to include the entire dataset, spanning ages 20 to 80, to identify reliable indicators for distinguishing young, middle, and old adults in cases of overlapping groups remains an important area for future research. Nevertheless, the findings address significant limitations of traditional visual age estimation methods, which often relied on broad categorizations like young, middle, and old adults due to extensive overlaps between age phases. This research provides a foundation for more refined and precise age estimation, advancing methodologies beyond the constraints of earlier approaches.

### Shape model comparison

The results observed in the YA group are consistent with those of previous studies, demonstrating that younger age groups undergo more pronounced morphological changes and are easier to distinguish than other age groups^13,15,17,18^. However, the transition from the 20 s to the 30 s reveals distinct patterns of change between males and females, highlighting a significant sex influence beyond the effects of aging. The extreme, but statistically significant extension on the center of the ventral margin in males happened in place where the superior margin of the suspensory ligament that supports the base of the body of the penis attached to the pubic symphysis^43^. This extension is presumed to be associated with the greater tension experienced by the suspensory ligament, owing to the protrusion of the external male sex organs. However, particularly in young adults, further anatomical studies are necessary to examine the correlation between suspensory ligaments and aging. With respect to females, morphological changes were not statistically significant, which may be attributed more to fluctuating individual differences due to external factors, such as pregnancy, rather than to the natural aging process. South Korean women are known to experience their first childbirth at an average age of 33.5 years, with the highest childbirth rate occurring in their 30 s^44^. Additionally, pregnancy and childbirth in females can result in changes in pelvic floor muscles, such as weakening and sagging^45–47^. As some of the pelvic floor muscles are connected to the pubic bone^47^, it is plausible to speculate that the state of maternity could have influenced the morphology of the pubic symphyseal surface within this specific age range. However, since the pregnancy and childbirth statuses of the subjects in this study were unknown, it would be reasonable to investigate age-related changes reflecting this status in future research. Lastly, while males and females exhibited similar patterns of morphological changes from their 30 s to 40 s, there were subtle differences in the exact location of these changes. In the age group from their 30 s to 40 s, changes in males occurred around the origins of the rectus abdominis, pyramidalis, and gracilis muscles, whereas in females, they occurred near the origin of the adductor longus muscle^48–50^. As suggested in previous studies, variations in muscle use may influence the morphology of the pubic symphyseal surface^21^. However, research that specifically examines the primary muscles utilized by males and females within this age group, has not yet been carried out, therefore further investigation is necessary.

A significant observation in the MA group was that males in their 50 s and females in their 40 s experienced a sudden surge in the rate of change. Moreover, age-related bone loss generally begins around the age of 35–40 years in both males and females and these physiological changes could account for the noticeable morphological changes^51–53^. However, the greater extent of change in females compared to that in males can be attributed to the pronounced bone loss experienced by females from their 40 s to the early 50 s due to the postmenopausal state, whereas males undergo gradual age-related bone loss throughout their lives^51,52,54^. Furthermore, it is noteworthy to review the differences in the patterns of change between males and females. The changes observed in males align with the established aging process of the pubic symphyseal surface, which is characterized by surface smoothing^13,17^. In contrast, the females manifested a pattern of narrowing width, which contradicts the results reported by Lottering et al., who observed an increase in the maximum width of the pubic symphyseal surface with aging in an effort to uphold mechanical strength^55^. However, given that changes in females are not statistically significant, caution should be exercised when utilizing the results.

The results of the OA group in this study challenge the results reported by Kotěrová et al., which suggested that age could still be significantly distinguished even in individuals over the age of 50^18^. In our study, it was demanding to identify statistically significant morphological changes in both males and females over the age of 60. Thus, the morphological changes observed in individuals over 60 years of age cannot be considered a representation of the aging pattern across the entire population but rather resemble irregular variations due to individual features^16,17,56,57^. The morphological changes observed in females were particularly important. Despite not achieving statistical significance, the moderate changes observed in females suggest greater individual variability among females of advancing age compared to males. This finding contradicts previous results suggesting that females exhibit less individual morphological variability in the pubic symphysis than males^13^. As mentioned in the YA and MA groups, females are more likely to experience physiological events such as childbirth and menopause, which can influence the morphology of the pubic symphysis^45,58^. Additionally, the pelvic floor muscles connected to the pubic symphysis are known to undergo more age-related changes in females than in males^59^. Furthermore, sagging is known to increase with age, regardless of pregnancy and childbirth status^60^. Therefore, considering the results of this study, along with the hormonal and surrounding muscle aging patterns, females may exhibit greater morphological variability with advancing age.

### Shape-based classification

In the YA group, it was necessary to examine transitions from the 20 s to 30 s and from the 30 s to 40 s separately because of the differences in significant shape variations. The most critical shape variation for distinguishing between males in their 20 s and 30 s was the ventral-dorsal width. This aligns with previous research, suggesting an increase in the superior-inferior and ventral-dorsal lengths of the symphyseal surface with age^55^. However, apart from the transition of males in their 20 s to 30 s, width was not prioritized as a decisive factor in other transition groups. This suggests that width alone may not be sufficient for age classification but might be limited to distinguishing adolescents and young adults in their twenties from other age groups among specimens identified as male. Additionally, the second most important factor for distinguishing between males in their 20 s and 30 s was the curvature of the margin, which was in line with findings indicating a transition of the symphyseal surface to a rounded shape after the age of 30^16^. This factor was identified as the most important shape variation when distinguishing between females in their 20 s and 30 s, with less significance, suggesting greater individual variation in shape within the age group. Lastly, both males and females prioritized the minor notch-like feature on the upper one-third of the ventral border when distinguishing between the 30 s and 40 s age groups. This result corresponds to the description made in Phase IV of the Suchey and Brooks method, which noted a *hiatus* on the upper ventral rim, and is consistent with subsequent geometric morphometric studies^7,11,61^. However, previous studies either suggested a wide age range for the appearance of the notch or identified it as a feature occurring in older adults without specifying a particular age group. This study did not exclusively determine age groups. However, considering the results alongside those of previous research, the absence of this feature persisted until the 30 s, with its emergence occurring in the 40 s. To date, there has been no discussion on the underlying cause of this phenomenon. However, recent studies on the morphology of the four ligaments surrounding the pubic symphysis have revealed that the area where the notch forms overlaps with the superior and anterior pubic ligament^62^. Hence, ligament overlaps may contribute to notch formation; however, biomechanical validation of this hypothesis is necessary in future research.

In MA and OA, significant shape variations distinguishing the age groups primarily involved complex surface textures and presented challenges for standardized descriptions. This explains why computational methods analyzing subtle surface details through algorithms can enhance age estimation accuracy^16,56^. However, when previous studies calculated the morphological changes of the surface as bending energy, the energy scores were overestimated owing to irregularities of the pubic symphyseal surface. This leads to an underestimation of the age of the older individual^17^. Therefore, analyzing the patterns of surface shape variation obtained in this study could help rectify such errors in future research. Moreover, more complex surface shape variations were observed in females than in males. Previous studies mainly focused on males or analyzed mixed-sex samples^16–18^. Although mixed-sex results may be preferable when sex cannot be determined, if sex is identifiable, separate estimation methods for each sex should be applied, underscoring the importance of sex-specific estimation approaches.

## Conclusion

This study addressed significant limitations in computational approaches for age estimation of the pubic symphysis by conducting a detailed analysis of age-related morphological changes. Through SSM, the morphological changes associated with aging on the pubic symphyseal surface were visualized, and significant morphological features for age differentiation were identified.

However, the limitations that warrant further research are evident. When extracting significant shape variations for age classification, PCA was limited to only two successive age categories, making it challenging to estimate the age at which the pubic symphysis from various age groups is intermixed. Although sexual dimorphism is evident, the lack of three-dimensional analyses underscores the need for further investigation in this domain. For female subjects, in particular, considering their medical history, including factors such as pregnancy, childbirth, and menopause, could enhance the overall accuracy in detecting statistically significant morphological changes. Lastly, as this study only focused on the Korean population, further research dealing with various population groups would be advisable to gain a deeper understanding of the morphological aging processes on the pubic symphyseal surface.

Despite these limitations, the complexity of age-related changes in the pubic symphysis has been underscored by these findings, contributing to the advancement of forensic anthropology and offering valuable insights into improving age-at-death estimation methods.

## Electronic supplementary material

Below is the link to the electronic supplementary material.

Supplementary Material 1

## Data Availability

The datasets used and/or analyzed during the current study are available from the corresponding author upon reasonable request.
