# Supplementary material for: Age-related morphological changes of the pubic symphyseal surface: using three-dimensional statistical shape modeling
Source: Sci Rep. 2025 Jan 2;15:494. doi: 10.1038/s41598-024-84168-8 (PMC11696140; doi:10.1038/s41598-024-84168-8)
Supplement: Supplementary file 1 — Supplementary Material 1 [file 41598_2024_84168_MOESM1_ESM.pdf]

Supplementary Figure 1

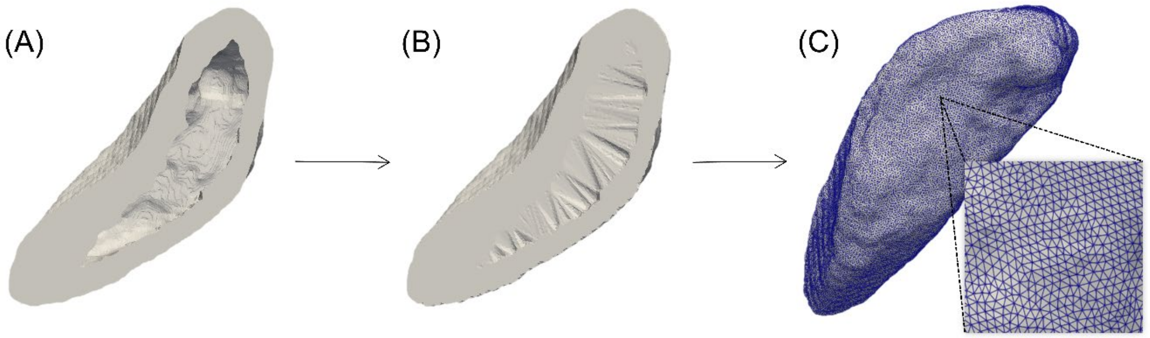

Preprocessing involves removing unnecessary bone meshes generated during the data acquisition and re-meshing the symphyseal surface data. This process was carried out using MeshLab (version 2022.02) and Meshmixer, then converting STL data to PLY using the Python VTK library (A). First, various factors potentially affecting shape analysis were eliminated using MeshLab's select faces in the rectangular region, fit plane to the selection, ambient occlusion, select faces by color, and delete selected face and vertices filters. After the application of filters, only the outer shell of the pubic symphysis data remained (Fig. 1a). Next, using the solid function of Meshmixer, hollowed-out areas were filled in water-tight to remove unnecessary elements inside that could affect analysis (B). Finally, MeshLab was used again to smooth the symphyseal surface data using screened Poisson surface reconstruction, isotropic explicit re-meshing, remove duplicate vertex and face filters. The last process not only smoothed but also redistributed vertices to regularize the topology of the data (C).

Supplementary Figure 2

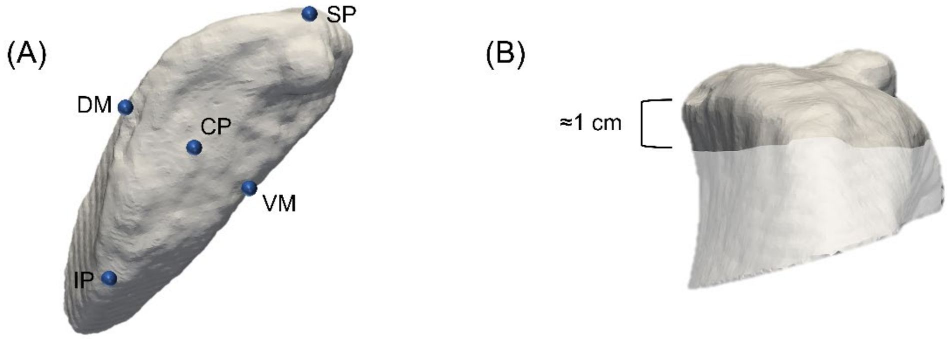

Shapes oriented in different positions and directions need to be aligned. If not, the positional or directional differences could be mistaken for morphological variability, leading to a failure to capture the shape variations. The five landmarks are as follows: in anatomical position, the midpoint of the dorsal margin (DM) and ventral margin (VM), the superior point (SP) and inferior point (IP) of the symphyseal surface, and the central point (CP) where lines connecting DM to VM and SP to IP intersect.

Supplementary Figure 3

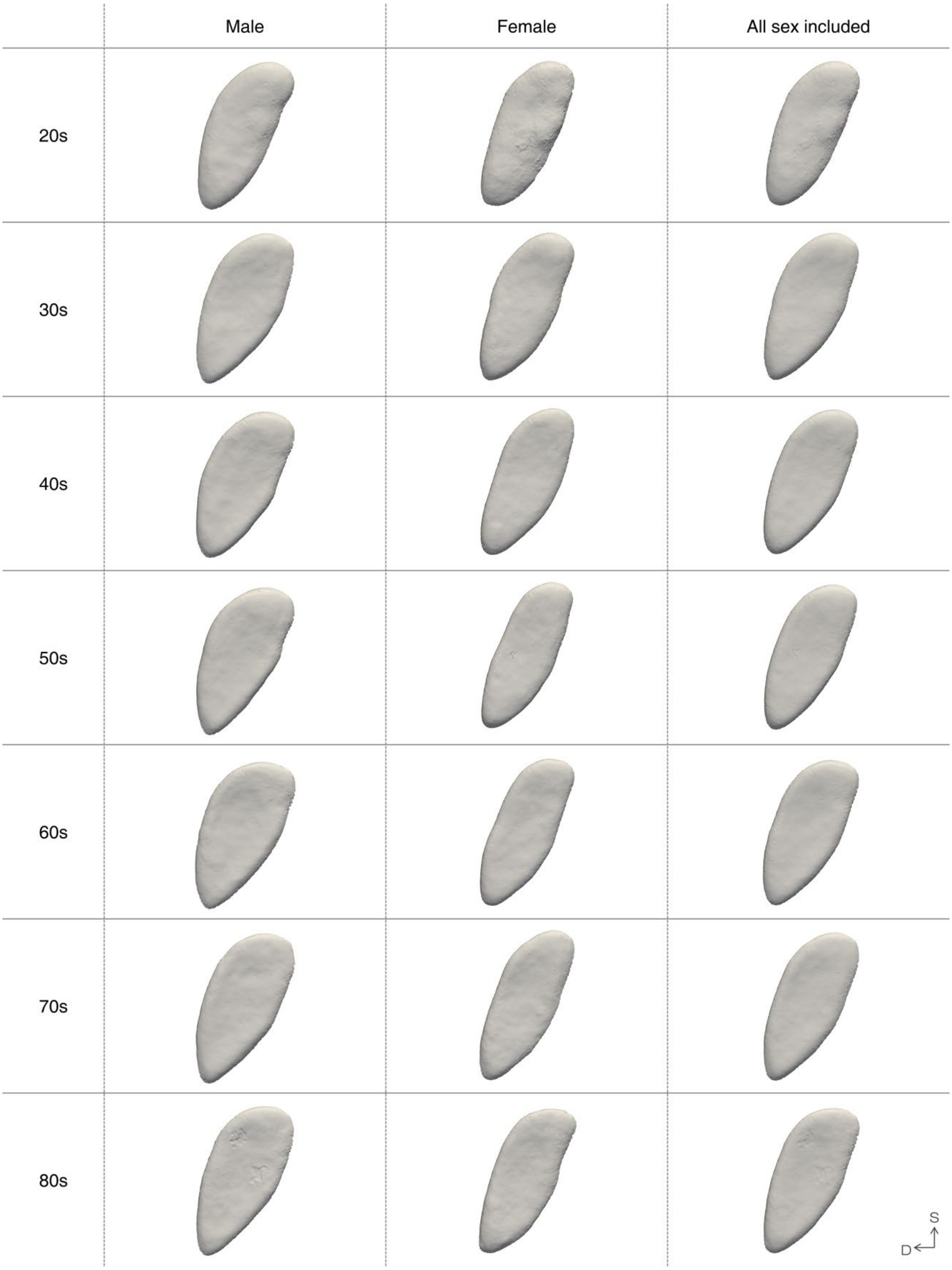

Mean pubic symphyseal surface model for each age and sex groups. In the orientation, ‘D’ stands for dorsal, and ‘S’ stands for superior.

Supplementary Table 1

Compared age groups	Algorithm model with highest accuracy		
	Male	Female	Total
20s → 30s	Logistic Regression Kernel	Gaussian Naive Bayes	SVM Kernel
30s → 40s	Efficient Logistic Regression	Bagged Tree	Efficient Logistic Regression
40s → 50s	Bagged Tree	Bagged Tree	Kernel Naive Bayes
50s → 60s	Logistic Regression Kernel	Fine Tree	Coarse Tree
60s → 70s	Binary GLM Logistic Regression	Medium Neural Network	Medium KNN
70s → 80s	Binary GLM Logistic Regression	Binary GLM Logistic Regression	Three-dimension KNN

Algorithm model with highest accuracy in each age groups.

Supplementary Table 2

Compared age groups	Explained variance (%)					
	Male		Female		Total	
20s → 30s	39.5	20.4	8.2	6.5	17.0	0.1
30s → 40s	12.0	8.4	4.1	1.7	3.2	7.4
40s → 50s	3.3	0.4	2.4	≈ 0.0	≈ 0.0	0.3
50s → 60s	5.2	0.4	3.4	0.8	4.2	≈ 0.0
60s → 70s	1.4	2.0	1.4	0.5	1.1	0.1
70s → 80s	0.4	5.0	0.1	0.4	1.4	≈ 0.0

Explained variance of significant principal components of each age group. Left is the explained variance of the most significant principal component and right is the following significant component.
